# Lineage-associated small inversions disrupt *dosT*, *dnaE2*, and a promoter-adjacent region in some *Mycobacterium tuberculosis* isolates

**DOI:** 10.1128/spectrum.02469-25

**Published:** 2026-08-10

**Authors:** Nikhil Bhalla, Nikita Goswami

**Affiliations:** 1 Department of Medical Epidemiology and Biostatistics, Karolinska Institutet 27106, Stockholm, Sweden; 2 Translational Health Group, International Centre for Genetic Engineering and Biotechnology, New Delhi, India; 3 Department of Biochemistry, University of Delhi South Campus 93081, New Delhi, India; Ross University School of Veterinary Medicine, Basseterre, Saint Kitts and Nevis; Salahaddin University-Erbil, Erbil, Iraq; Universite Laval, Quebec City, Quebec, Canada; Indian Institute of Technology Jodhpur, Jodhpur, Rajasthan, India

**Keywords:** inversions, structural variations, TB, fusion proteins, domain switching

## Abstract

Large molecular inversions in the genome of *Mycobacterium tuberculosis* (Mtb) due to factors like the presence of insertion sequences and transposases are widely known. However, smaller inversions within coding sequences and non-coding control elements are rarely reported. The present study aims to identify inversions and their potential impact on Mtb biology in a lineage-specific manner. Structural variants (SVs) could only be detected by long reads. For this, we simulated long reads by *de novo* assembling the short-read sequencing data sets and subsequently aligned representative strains from each lineage using the Progressive Mauve algorithm. Independently, long-read sequencing from the Pacific Biosciences platform was acquired and analyzed using the structural variant identification method. Variants were merged, and Fisher’s exact test was carried out to identify the inversion association with lineages. To visualize deoxyribonucleic acid (DNA) features, the DNA-features-viewer tool was used. Simulated reads from short-read sequencing gave indications of lineage (L)-specific inversions. The long-read sequencing approach led to the identification of seven unique inversions: two positively associated with L1, one positively associated with L3, two negatively associated with L4, and two positively associated with L3 but negatively associated with L4 (*P* < 0.05). The inversions encompassed primarily non-essential genes like *sdaA*, *dosT*, Rv2026c, *dnaE2*, Rv1341, Rv1342, and *lprD*. An interesting inversion was observed in the upstream control element of *purB* and Rv0776c. The study sheds light on small inversions that may be causing alterations in expression, formation of fusion genes, and nonsense mutations that may have a role in lineage-specific phenotypic changes.

**IMPORTANCE:**

The role of mutations like SNPs and INDELs and their association with drug resistance is well known in *Mycobacterium tuberculosis* (Mtb). However, structural variations, especially inversions, are largely overlooked and unreported. In this paper, publicly available whole-genome sequencing datasets from Illumina and Pacific Biosciences–Oxford Nanopore Technologies platform have been used to detect inversions and report seven unreported Mtb lineage-specific small inversions.

## OBSERVATION

Genetic inversions, a type of structural polymorphism, occur when a part of the genome is flipped in reverse orientation. In other words, when a region of the genome becomes reverse complementary relative to the rest of the genome, it is what is called a true inversion (1–3). Inversion-type structural variations are widely studied in organisms higher in the hierarchy, such as animals, plants, insects, and unicellular eukaryotes (4–6). In the case of bacteria, isolated literature suggests the presence of inversion events, such as *rrnD-rrnE*, which was observed in a laboratory strain of *Escherichia coli* K12, and then the same inversion was reinverted years later. These events can potentially have a drastic impact on the phenotype of the bacteria, such as a growth disadvantage (7). Similarly, such events are reported in *Clostridium* and are mediated by recombinases encoded by RecV and are of larger sizes: 5–50 kbp (8). The inversions are also reported in promoter regions, altering expression levels, resistance development, and aiding adaptation in gut microbiota (9). Inversions in bacteria can present themselves within the coding region, leading to “domain switching” and alterations in the binding target proteins. Such events are reported in *Bacteroides fragilis* and *Streptococcus pneumoniae* (10–12). In *Mycobacterium*, owing to the presence of recombinases, large-scale intragenomic recombination events are inevitable, resulting in duplication, deletions, and inversions. In the event of duplication, the transposon can fit in the genome in either direction, potentially causing inversion (13, 14). However, in the majority of *Mycobacterium tuberculosis* (Mtb)-specific studies, large inversions caused by mobile genetic elements are reported (13, 15, 16).

The present study shows signatures of inversions through short-read sequencing data and actual seven small 197–681 bp inversions through the analysis of long-read sequencing data sets. Interestingly, despite being small, some of the inversions seem to disrupt more than one gene at a time. These inversions may have a role in lineage-specific prognosis differences.

## MATERIALS AND METHODS

### Data acquisition

Whole-genome sequencing (WGS) data sets were downloaded from the European Nucleotide Archive (ENA) in the form of fastq format. Some WGS data sets from the Pacific Biosciences (PacBio)–Oxford Nanopore Technologies (ONT) platform were not available for download in the FASTQ format from ENA. Such data sets were downloaded from the Sequence Read Archive (SRA) in SRA format. The files in SRA format were converted to fastq using the fastq-dump command from the SRA toolkit. The Bio Projects and lineage composition for short-read and long-read sequencing data sets are shown in Table S1.

### Detecting inversions using assemblies made using short-read sequencing

The short reads were first adapter-trimmed using bbduk.sh. Adapter-trimmed reads were subjected to Shovill (v1.1.0) (assembler: megahit) for *de novo* assembly and polishing (17, 18). The consistency of assemblies was improved using RagTag prior to downstream structural variation analysis. The complete *Mycobacterium tuberculosis* H37Rv genome (RefSeq accession: NC_000962.3) was used as the reference genome. RagTag was run in correction mode using the “ragtag.py correct” command. This correction step was included to reduce the effects of potential assembly inconsistencies before generating locally collinear block (LCB)-based inversion and translocation matrices. The corrected assemblies generated by RagTag were subsequently subjected to WGS-based drug resistance and lineage detection using tb-profiler (v6.6.3) (19). The assemblies were subjected to Parsnp (v1.2) for the determination of distances (20). The tree from Parsnp and tb-profiler output were used to generate an annotated dendrogram using the interactive tree of life (iTOL). A phylogenetic free distance matrix was created, and 10 replicates per lineage were selected for analysis with ProgressiveMauve (v20150226) with and without the assumption of collinearity (21). Apart from 10 replicates per lineage, a representative complete *Mycobacterium tuberculosis* H37Rv genome (RefSeq: NC_000962.3) was also included in the Progressive Mauve alignment. The alignment backbone output was parsed to identify LCBs, and inversion-associated LCB orientation values were converted into binary variables, where positive and negative orientations were coded as 1 and 0, respectively. The resulting binary inversion matrix was subjected to categorical principal coordinate analysis (PCA), using the FactoMineR R package (22). The first two categorical principal coordinate (cPC) axes were plotted to visualize lineage-associated clustering of inversion-derived structural variation patterns.

### Detecting inversions using long-read WGS Mtb sequencing data sets

The single-ended long reads were filtered using seqkit (v2.3.0), and only reads with length > 500 bp were retained (23). The reads were subjected to *de novo* assembly using Rapid Assembler for Viral and Eukaryotic Nucleotides (RAVEN) (v1.6.1), and these were used for WGS-based drug resistance and lineage detection purposes with tb-profiler (19, 24). Long sequencing reads were subjected to mapping with Minimizer-based Mapper 2 (minimap2) (v2.26-r1175) using the reference genome (RefSeq: NC_000962.3) (25). The alignment files were sorted and subsequently sorted using Sequence Alignment/Map tools (samtools) (26). The Binary Alignment/Map (BAM) files were used as input for structural variant identification method (SVIM) (v2.0.0) for the detection of inversions (27). The sample IDs and variants in variant call format (VCF) files were named and sorted using binary call format tools (bcftools), sort and reheader options (v1.19) before collating into a multi-sample VCF. Survivor (v1.0.7) was used for merging single-sample VCF files into a single file (28). For merging, the maximum distance between breakpoints to be considered as a structural variant was set to 1,000; the minimum presence of the inversion should be in at least 10 samples, distance-based SV size estimation and merging were enabled, and the minimum structural variant (SV) = 50 bp. The VCF file was converted to a table and subsequently parsed in Microsoft Excel (MS-Excel) and R for data analysis and visualizations.

### Computational resources

All analyses were performed on a homegrown bioinformatic data analysis setup running Windows Subsystem for Linux 2 (WSL2) on AMD Ryzen 5600G with 64 GB of random access memory (RAM) and another running on Intel i5-8265U with 24 GB RAM. The jobs were distributed and parallelized between the two computers using GNU’s Not Unix (GNU) parallel (v20160622). The tools used in the study are open-source and can be downloaded and installed from GitHub and Anaconda.

### Statistical analysis

The FactoMineR package was used for determining the cPC for each sample. For the small-scale association analysis, because of the small sample size, Fisher’s exact test was deemed appropriate and carried out in R. Benjamin Hochberg’s (BH) correction of *P* values was also carried out. Inversions showing *P* < 0.05 were considered significant and are reported in the present study.

## RESULTS

### Short-read polished assemblies indicated the presence of inversions

**Fig 1:**
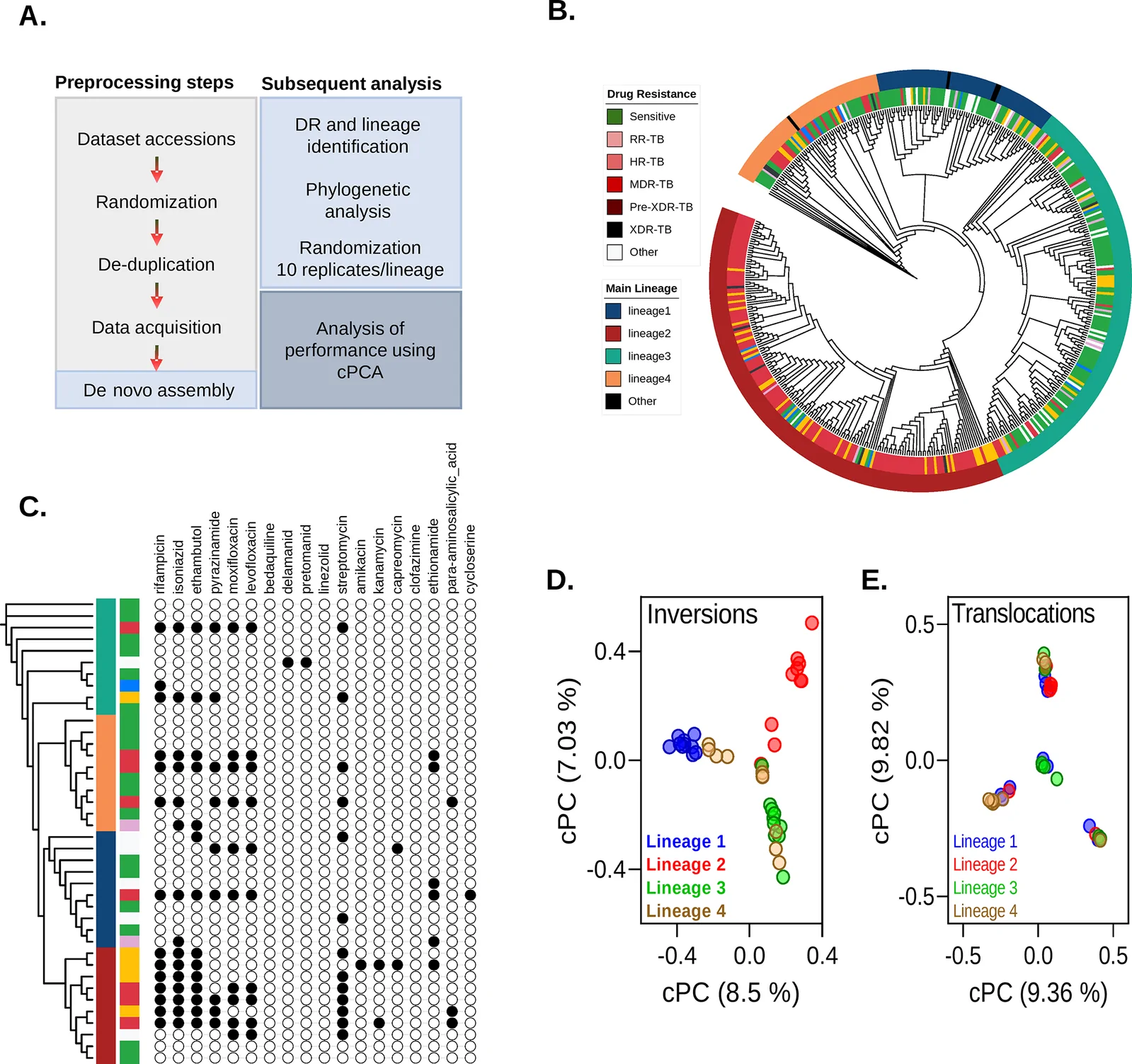
Short-read sequencing technologies indicated the presence of lineage-specific inversions. (**A**) Strategy of data selection, acquisition, and processing. (**B**) Multi-genome alignment of 467 corrected assemblies was carried out using Parsnp, and tb-profiler was used for determining drug resistance and lineages. (**C**) Pairwise genomic distances were computed using Mash, which is based on k-mer similarity between assemblies. The resulting distance matrix was used for generating a neighbor-joining tree. (**D**) Inversion data in the form of LCB were examined, and those showing negative and positive values were converted to binaries, 0 and 1, respectively. (**E**) Translocations matrix was generated using the estimated midpoint in LCB. If the midpoint deviated, it was considered 1; otherwise, it was considered 0. The matrices in binary were subjected to cPCA, aka multiple correspondence analysis, using the R package FactoMineR. The data were replotted in GraphPad Prism. Note: the trees and associated data sets were visualized in the iTOL online platform. RR, rifampicin resistant; HR, isoniazid resistant; MDR, multidrug resistant; pre-XDR, pre-extensively drug resistant; cPCA, categorical principal coordinate analysis.

From Illumina-based short sequencing reads deposited in SRA with BioProjects PRJEB41116 and PRJNA879962, a subset of 500 assemblies was selected. Randomization did not yield unique numbers; as a result, there were some duplicate assemblies that had to be removed. Finally, 467 WGS data sets were acquired from the European Nucleotide Archive (Table S1). Based on N50 (2,817,734 ± 1,888,117), genome fraction (98.9% ± 11.5%), and total length (4,403,718 ± 513,257) from *de novo* assembly quality control analysis, poor-quality samples were identified and eliminated. WGS-based lineage detection was also correlated with samples and is included in the *de novo* assembly statistics table of short-read data sets, shown in Table S2. The subsequent analysis was done with a set of 467 pure lineage and high-quality samples. A schematic representation of the procedure followed for analysis is shown in Fig. 1A. Phylogenetic clustering of 467 samples showed lineage-specific clustering, and higher prevalence of drug resistance in lineage 2 isolates was observed (Fig. 1B). A subset of 40 isolates (10 samples per lineage) was subsequently processed for alignment using the ProgressiveMauve algorithm. The corrected *de novo* assemblies that were aligned using ProgressiveMauve showed distorted patterns. Alignment-free clustering based on the mash distance matrix also clustered samples according to lineages. Again, lineage 2 samples from this subset showed resistance to a greater number of drugs, and only 2 of 10 lineage 2 isolates were sensitive (Fig. 1C). Even though ProgressiveMauve results were not conclusive, the LCB matrices built using the approaches mentioned in the methodology section showed clustering on categorical PCA according to lineages as far as inversions were concerned (Fig. 1D). A similar approach for translocations clustered samples into four subclusters. However, in the case of translocations, each cluster contained isolates from varying lineages (Fig. 1E). This indicated that translocations are probably associated with an unknown factor, unlike in the case of lineages.

### SV determination from long reads

**Fig 2:**
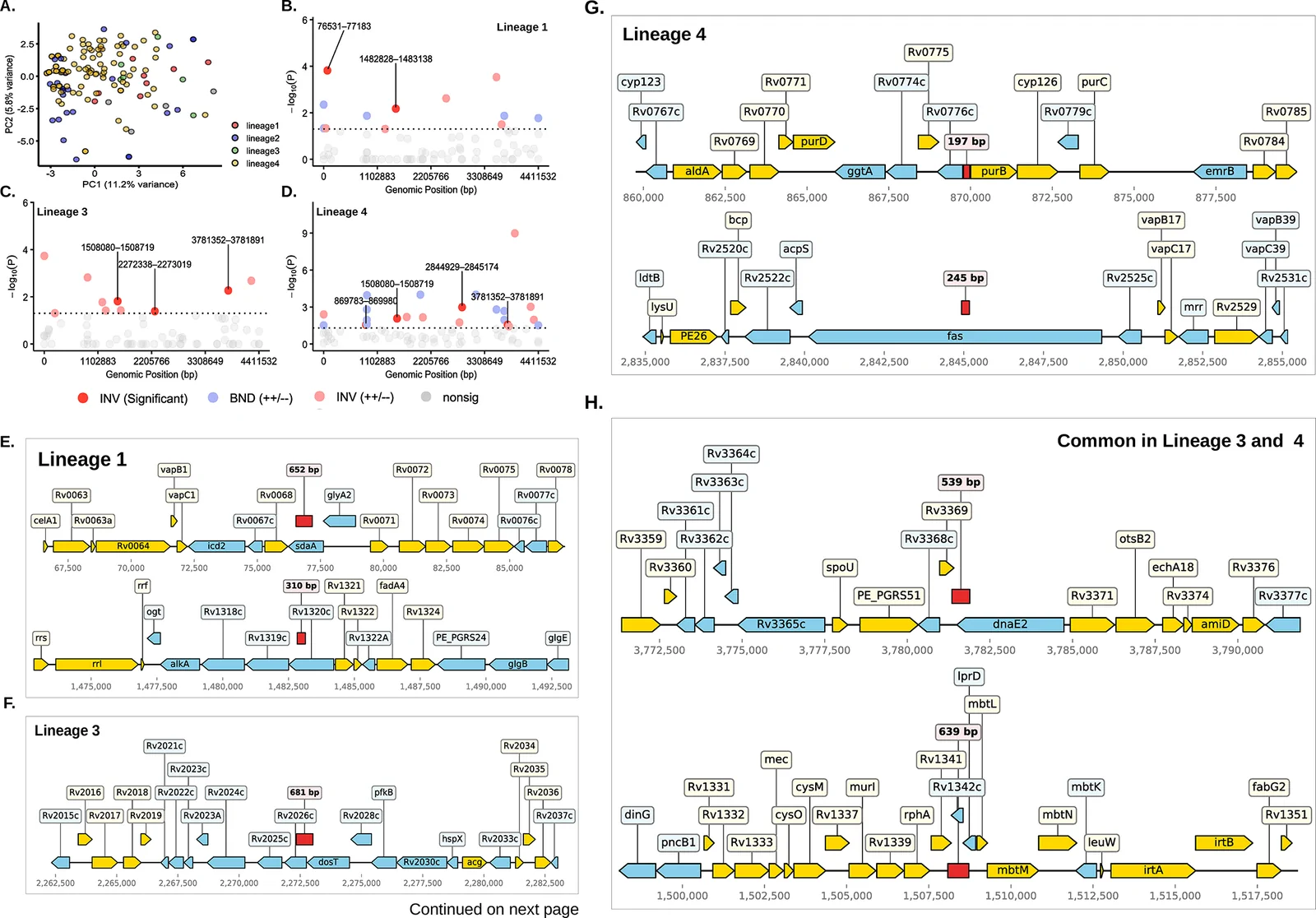
Genetic inversions determined using PacBio reads showed a lineage-specific distribution in the Mtb coding sequence (CDS) and non-coding regions. The long reads were assembled using minimap2 on the NC_000962.3 reference genome, and SVIM was used for calling variants. The VCF files from SVIM were modified using BCFtools to add sample ID and sorting. Finally, the files were merged with SURVIVOR, and the merged multi-sample VCF file was converted to a table. Independently, the reads were also *de novo* assembled using RAVEN, and the resulting assemblies were used as input for tb-profiler. Lineages determined using tb-profiler were used for grouping samples for association analysis. (**A**) The matrix of INVs and BNDs was used as input for determining their principal coordinate. (**B–D**) For determining the association of specific inversions, Fisher’s exact test was performed. INVs with breakpoints on both strands (+/−) were considered true gene inversion events and are labeled. The associations were visualized as −log10P vs genome coordinates as Manhattan plots (RefSeq: NC_000962.3). (**E–H**) The significant INVs with breakpoints on both strands found in lineages 1, 3, and 4 are visualized on the genome of Mtb to understand their location and their potential impact on Mtb strains of particular lineages. Inversions are highlighted in red, forward-strand genes in yellow, and reverse-strand genes are visualized in blue. INV, inversions; BND, BreakEND; nonsig, non-significant.

Long-read WGS data sets of 237 PacBio data sets originating from cultures were identified using the metadata and downloaded. A subset (*n* = 182) of data sets could be assembled successfully into fewer contigs. The others that could not be assembled were either due to poor quality (<500 bp average read length) or limited computational resources. Lineage determination using tb-profiler revealed a major fraction of lineage 4 (*n* = 113), followed by lineage 2 (*n* = 6), and mixed lineages (*n* = 28). The data sets representing mixed lineages were eliminated from subsequent analysis, except lineage 2/4 samples. Inversion (INV) and BreakEND (BND) were filtered for PCA analysis, which showed subtle clustering on the graph, indicating the presence of fewer INVs and BNDs between lineages (Fig. 2A). Inversions with breakpoints on both strands were considered as true inversions, and those that had breakpoints only on one of the strands were, despite being significant, not considered true inversions. In lineage 1, a set of two inversions, 652 bp and 310 bp, were observed with coordinates 76,531–77,183 (raw *P* < 0.0001; BH *P* < 0.016) and 1,482,828–1,483,138 (raw *P* < 0.01; BH *P* = 0.08), respectively (Fig. 2B) (RefSeq: NC_000962.3). In lineage 3, a set of three inversions of 639 bp, 681 bp, and 539 bp were observed with coordinates 1,508,080–1,508,719 (raw *P* = 0.01; BH *P* = 0.1); 2,272,338–2,273,019 (raw *P* = 0.04; BH *P* = 0.1), and 3,781,352–3,781,891 (raw *P* = 0.005; BH *P* = 0.09) (Fig. 2C) (RefSeq: NC_000962.3). Lineage 4 was negatively associated with four inversions of 197 bp, 639 bp, 245 bp, and 539 bp with coordinates 869,783–869,980 (raw *P* = 0.02; BH *P* = 0.2), 1,508,080–1,508,719 (raw *P* = 0.008; BH *P* = 0.1), 2,844,929–2,845,174 (raw *P* = 0.001; BH *P* = 0.02), and 3,781,352–3,781,891 (raw *P* = 0.02; BH *P* = 0.2), respectively (Fig. 2D) (RefSeq: NC_000962.3). The observed lineage inversions in lineage 1 of 652 bp and 310 bp were within the genes *sdaA* and Rv1320c (Fig. 2E). Another inversion of 681 bp in lineage 3 was within but at the latter end of the coding region of *dosT* and within Rv2026c. Both these genes are reverse in direction. Probably this disorientation is causing a gene fusion or, more likely, disruption of both (Fig. 2F). A set of two inversions negatively associated with lineage 4 included a 197 bp inversion in the upstream control region of the two genes oriented away from each other (Rv0776c and *purB*), probably having an impact on their expression. The other 245 bp inversion is within the centroid of the *fas* gene (Fig. 2G). A set of two inversions positively associated with lineage 3 and negatively associated with lineage 4 included a 539 bp inversion encompassing the latter end of Rv3369 and the latter end of *dnaE2*, and a 639 bp inversion disrupting three relatively small genes oriented in opposite directions in lineages 3 and 4. The three genes included Rv1341 (forward), Rv1342 (reverse), and *lprD* (reverse) (Fig. 2H). Some of these inversions were also found in samples of mixed lineages (lineage 2 and lineage 4), and these included 869,783–869,980, 1,508,080–1,508,719, and 2,844,929–2,845,174 (Fig. S1). Due to low sample size, true inversions were not observed in lineage 2 isolates (Fig. S1). A summary of inversions is included in Table 1.

**TABLE 1:** Summary of statistically significant inversions^*a*^

S. no.	Lineage	Start	End	Raw *P*	BH *P* value	Genomic region	Carriers in lineage	Freq in lineage (%)	Carriers in others	Freq in others (%)	Overall carriers	Overall freq (%)	Association
1	Lineage 1	76,531	77,183	1.52E−04	1.69E−02	*sdaA* (Rv0069c)	8/9	88.9	56/226	24.8	64/235	27.2	Positive
2	Lineage 1	1,482,828	1,483,138	6.68E−03	8.03E−02	Rv1320c	6/9	66.7	50/226	22.1	56/235	23.8	Positive
3	Lineage 3	1,508,080	1,508,719	1.54E−02	1.40E−01	Rv1341, Rv1342c, *lprD* (Rv1343c)	3/6	50	21/229	9.2	24/235	10.2	Positive
4	Lineage 3	2,272,338	2,273,019	4.09E−02	1.87E−01	Rv2026c, *dosT* (Rv2027c)	3/6	50	31/229	13.5	34/235	14.5	Positive
5	Lineage 3	3,781,352	3,781,891	5.38E−03	9.92E−02	Rv3369, *dnaE2* (Rv3370c)	5/6	83.3	57/229	24.9	62/235	26.4	Positive
6	Lineage 4	869,783	869,980	2.79E−02	2.29E−01	CDS flanked by Rv0776c (upstream) and purB (Rv0777) (downstream)	8/112	7.1	21/123	17.1	29/235	12.3	Negative
7	Lineage 4	1,508,080	1,508,719	8.46E−03	1.19E−01	Rv1341, Rv1342c, *lprD* (Rv1343c)	5/112	4.5	19/123	15.4	24/235	10.2	Negative
8	Lineage 4	2,844,929	2,845,174	1.03E−03	2.91E−02	fas (Rv2524c)	15/112	13.4	39/123	31.7	54/235	23	Negative
9	Lineage 4	3,781,352	3,781,891	2.71E−02	2.26E−01	Rv3369, *dnaE2* (Rv3370c)	22/112	19.6	40/123	32.5	62/235	26.4	Negative

^*a*^ Inversions encompassed CDS and control elements disrupting or potentially altering their expression, respectively. The complete *Mycobacterium tuberculosis* reference genome (RefSeq: NC_000962.3) was used. *P* values were determined using Fisher’s exact test to analyze their lineage associations. “Start” and “End” give the inversion breakpoint coordinates in base pairs relative to the *M. tuberculosis H37Rv* reference genome (RefSeq: NC_000962.3), and genomic region indicates the overlapping gene or control element with its Rv locus tag. For each inversion, “Carriers in lineage” and “Carriers in others” report the number of isolates carrying the inversion out of the total isolates analyzed within the indicated lineage and across all other lineages combined, respectively, while “Overall carriers” gives the corresponding count for the full data set. “Freq in lineage (%),” “Freq in others (%),” and “Overall freq (%)” express these counts as the respective percentages. “Association” denotes the direction of the lineage relationship, with positive indicating enrichment and negative indicating depletion of the inversion within the indicated lineage. S.no., serial number; CDS, coding sequence; Freq, frequency; raw *P*, uncorrected Fisher’s exact test *P* value; BH, Benjamini–Hochberg adjusted *P* value (false discovery rate).

## DISCUSSION

Structural variations in coding and non-coding regions can have far-reaching impacts on bacterial phenotype. In a recent study, a novel tool was developed that identified a structural variation in the virulence-associated ESX-5-encoding gene. In addition, they also genotyped 41,134 isolates and found association of some SVs with resistance to various first- and second-line drugs (29). It is common to disrupt the genes of interest in laboratory conditions, and it is routinely done. However, in natural conditions, pathogenic bacteria may also be doing the same on their own through mechanisms already known or through yet-to-be-deciphered mechanisms. By disrupting some genes and reorienting promoter regions to express genes that favor Mtb’s survival, inversions can cause changes in host-pathogen interactions, growth defects, and virulence. To identify inversions in the Mtb genome, short-read WGS data were assembled into longer contigs to simulate long reads; however, this approach did not yield conclusive results matching earlier recommendations and literature that for the identification of structural variations, long-read sequencing is necessary. In lineage 1, we found two inversions, both in CDS-*sdaA* and Rv1320c. SdaA is a non-essential iron-sulfur containing L-serine dehydratase and catalyzes serine deamination. Because of the inversion in *sdaA*, SdaA levels might be low or have alternate functions due to potential domain switching. Lineage 1 is an ancient lineage and is considered to be less virulent compared to modern Mtb lineages (30). Rv1320c encodes adenylyl cyclase, which was found to have an inversion in its CDS and is also known to be present in more virulent strains of Mtb (31). *dosT* encodes a two-component sensor histidine kinase, DosT. It was also found to have an inversion in its CDS. DosT has a role in sensing hypoxia and dormancy induction (32, 33). So, an inversion in *dosT* CDS in lineage 3, particularly, might be responsible for dormancy differences that are well-known in Mtb lineages. The same inversion was also found to encompass its neighboring gene Rv2026c, which encodes for the universal stress protein family protein and has a role in virulence, detoxification, and adaptation. Rv2026c is also known to get upregulated in stress conditions like oxidative stress. Rv2026c knockout strains are highly induced in hypoxic conditions, just like *dosT* (34). Probably, a cross-gene inversion between *dosT* and Rv3026c is leading to the generation of a fusion gene product with similar function. The role of Rv0776 is unknown; however, it is under the control of a non-coding region that probably controls its expression, which was found to be inverted and negatively associated with lineage 4, and another gene that flanks this potential promoter region is *purB*, which encodes an RNase-transformylase T, which has a role in the *de novo* purine salvage pathway (35). This reorientation of the promoter region flanked by two genes is interesting and requires more attention. A centroid-region inversion negatively associated with lineage 4 was observed in the *fas* gene that encodes fatty acid synthase Fas. The gene is relatively long, so a small inversion in its center may not be impacting its active site, as it is an essential gene. Its expression is known to be higher in strains other than lineages 2 and 4 (KZN605). The inversion might not have a role in expression level changes, but it might lead to the production of short-length proteins, which could not be detected, leading to the assumption that it has higher expression in lineage 2 and KZN605 (36). *dnaE2* encodes deoxyribonucleic acid (DNA) polymerase III (alpha chain) DnaE2 (DNA nucleotidyl transferase), so an inversion in the latter end might not significantly impact its DNA polymerase activity. It is important to note that DnaE2 is an error-prone DNA polymerase that is upregulated in response to drugs, leading to mutations in drug target genes, causing drug resistance (37). An inversion in the latter end of *dnaE2* of lineage 3 could be of interest. Another inversion positively associated with lineage 3 and negatively with lineage 4 encompassed three genes oriented in different directions: Rv1341 (encodes a conserved protein), Rv1342c (membrane protein), and *lprD* (encodes lipoprotein). The inversion might be causing an alteration in the expression of Rv1342c and leading to the generation of fusion genes of *lprD* and Rv1341.

The study sheds light on short 197–681 bp long inversions in CDS and non-coding regions that could have an impact on phenotypes, lead to the formation of fusion gene products, and alter expression in a lineage-specific manner. Moreover, future experimental validation studies are required to confirm the identified inversions and elucidate their precise contribution to *Mycobacterium tuberculosis* physiology, adaptation, virulence, and lineage-specific evolution. The primary objective of the study was to perform a pilot-scale genome-wide identification of lineage-associated small inversions (if any) using complementary short-read and long-read sequencing data sets. Functional validation through approaches such as polymerase chain reaction (PCR)-based confirmation, transcriptomic analyses, gene expression studies, targeted mutagenesis, and phenotypic characterization may be carried out by researchers having access to isolates and necessary facilities. We believe that, despite the absence of experimental validation, the systematic identification of lineage-associated small inversions using independent sequencing strategies provides important information pertaining to the evolution of this deadly bacterium.

### Conclusion

The current study provides an analysis of genomic inversions in *Mycobacterium tuberculosis* and highlights their potential as an underappreciated source of genetic evolution and lineage-specific phenotypic diversification. This study establishes a framework for the identification and validation of lineage-associated inversion events, by integrating *de novo* assembly-based analyses of short-read sequencing data with independent long-read sequencing and structural variant detection. From the analysis, we speculate that the identified inversions may influence gene expression by altering regulatory regions, disrupting coding sequences, facilitating the formation of fusion genes, and introducing nonsense mutations that can potentially affect overall gene function. Furthermore, the yet-to-be-identified inversions in the *M. tuberculosis* genome may affect genes involved in stress adaptation, DNA repair, metabolism, and regulatory processes.

## Supplementary Material

Reviewer comments

## Data Availability

All data generated are included in the manuscript. The study used publicly available WGS data sets in NCBI-SRA. The WGS data sets used in the study are provided in the supplemental material with BioProjects accession IDs in Table S1.

## SUPPLEMENTAL MATERIAL

Supplemental material spectrum.02469-25-s0001.docx Fig. S1; Tables S1 and S2.

OPEN PEER REVIEW reviewer-comments.pdf An accounting of the reviewer comments and feedback.

ASM does not own the copyrights to Supplemental Material that may be linked to, or accessed through, an article. The authors have granted ASM a non-exclusive, world-wide license to publish the Supplemental Material files. Please contact the corresponding author directly for reuse.
