# Supplementary material for: Lineage-associated small inversions disrupt dosT, dnaE2, and a promoter-adjacent region in some Mycobacterium tuberculosis isolates
Source: Microbiol Spectr. 2026 Aug 10;14(9):e02469-25. doi: 10.1128/spectrum.02469-25 (PMC13532295; doi:10.1128/spectrum.02469-25)
Supplement: Supplementary file 1 [file reviewer-comments.pdf]

ASM | Microbiology Spectrum

Lineage-associated small inversions disrupt *dosT*, *dnaE2*, and a promoter-adjacent region in some *Mycobacterium tuberculosis isolates.*

Nikhil Bhalla and Nikita Goswami

Corresponding Author(s): Nikhil Bhalla, Karolinska Institutet

Review Timeline:

Submission Date:	August 10, 2025
Editorial Decision:	March 12, 2026
Revision Received:	July 2, 2026
Accepted:	July 18, 2026

Editor: Felix Toka

Reviewer(s): Disclosure of reviewer identity is with reference to reviewer comments included in decision letter(s). The following individuals involved in review of your submission have agreed to reveal their identity: Fairuz Hassan Abdullah Tawgozy (Reviewer #1); Arijit Bhattacharya (Reviewer #2); Shankar Manoharan (Reviewer #3)

Transaction Report:

(Note: With the exception of the correction of typographical or spelling errors that could be a source of ambiguity, letters and reports are not edited. The original formatting of letters and referee reports may not be reflected in this compilation.)

DOI: https://doi.org/10.1128/spectrum.02469-25

Re: Spectrum02469-25 (Lineage-associated small inversions disrupt *dosT*, *dnaE2*, and a promoter-adjacent region in *Mycobacterium tuberculosis*.)

Dear Dr. Nikhil Bhalla:

Thank you for the privilege of reviewing your work. Below you will find my comments, instructions from the Spectrum editorial office, and the reviewer comments.

The authors should thoroughly revise the Methods section to provide full details of computational parameters, reference sequences used (e.g., for RagTag), and the workflow for detecting inversions, as well as clearly compare the performance of short-read versus long-read assemblies in identifying structural variants. They should also strengthen the Results and Discussion by explaining the PCA results, reporting lineage-specific inversion frequencies, and clarifying the potential biological significance of the detected inversions. Finally, the manuscript would benefit from improved clarity and structure (defining abbreviations, adding a formal conclusion, improving readability), and if feasible, adding experimental validation or at least stronger supporting evidence for the functional relevance of the reported inversions.

Please return the manuscript within 60 days; if you cannot complete the modification within this time period, please contact me. If you do not wish to modify the manuscript and prefer to submit it to another journal, notify me immediately so that the manuscript may be formally withdrawn from consideration by Spectrum.

Revision Guidelines
To submit your modified manuscript, log into the submission site at https://spectrum.msubmit.net/cgi-bin/main.plex. Go to Author Tasks and click the appropriate manuscript title to begin. The information you entered when you first submitted the paper will be displayed; update this as necessary. Note the following requirements:

• Upload point-by-point responses to the issues raised by the reviewers in a file named "Response to Reviewers," NOT in your cover letter.
• Upload a compare copy of the manuscript (without figures) as a "Marked-Up Manuscript" file.
• Upload a clean .DOC/.DOCX version of the revised manuscript and remove the previous version.
• Each figure must be uploaded as a separate, editable, high-resolution file (TIFF or EPS preferred), and any multipanel figures must be assembled into one file.
• Any supplemental material intended for posting by ASM should be uploaded with their legends separate from the main manuscript. You can combine all supplemental material into one file (preferred) or split it into a maximum of 10 files with all associated legends included.

For complete guidelines on revision requirements, see our Submission and Review Process webpage. Submission of a paper that does not conform to guidelines may delay acceptance of your manuscript.

Data availability: ASM policy requires that data be available to the public upon online posting of the article, so please verify all links to sequence records, if present, and make sure that each number retrieves the full record of the data. If a new accession number is not linked or a link is broken, provide Spectrum production staff with the correct URL for the record. If the accession numbers for new data are not publicly accessible before the expected online posting of the article, publication may be delayed; please contact production staff (Spectrum@asmusa.org) immediately with the expected release date.

Publication Fees: For information on publication fees and which article types are subject to charges, visit ourwebsite. If your manuscript is accepted for publication and any fees apply, you will be contacted separately about payment during the production process; please follow the instructions in that e-mail. Arrangements for payment must be made before your article is published.

ASM Membership: Corresponding authors may join or renew ASM membership to obtain discounts on publication fees. Need to upgrade your membership level? Please contact Customer Service at Service@asmusa.org.

The ASM Journals program strives for constant improvement in our submission and publication process. Please tell us how we can improve your experience by taking this quick Author Survey.

Thank you for submitting your paper to Spectrum.

Sincerely,
Felix Toka
Editor
Microbiology Spectrum

Reviewer #1 (Comments for the Author):

09.11.2025

Microbiology Spectrum
"Lineage-associated small inversions disrupt dosT, dnaE2, and a promoter-adjacent region in Mycobacterium tuberculosis.
Control no. Spectrum02469-25
Dear Author,
There is overall consistency throughout the manuscript. It is well designed. The discussion does not include references from (2025). It is novel and interesting, but there are several minor changes that should be considered to make it a perfect paper.
Please consider the following comments:
1. There are so many abbreviations without full form, when you mention something, you have to write it in full form first and then write the abbreviations in the next paragraphs.
2. Regarding the conclusion, you just mentioned it in the abstract, there is no detailed conclusion for the manuscript.
3. How long you need to do this work?

With regards,
Fairuz Hassan Abdullah, Ph.D.
Biotechnology and Molecular Biology Lecturer

Reviewer #2 (Comments for the Author):

In the article "Lineage-associated small inversions disrupt dosT, dnaE2, and a promoter-adjacent region in Mycobacterium tuberculosis", Bhalla N reports small inversions with possible impact in gene structure, expression linked to lineage specific phenotypic attributes. The report is intensive in identifying small inversions in various lineages. The methodologies are well implemented and well-drafted. The statistical analysis is also robust. The most elegant aspect of the study is analyzing short read and long read assemblies. There are points to be addressed as listed here in.

Comments:
1. The PCA for short and long read assembly data should be elaborated.
2. The significance of the study should have been highlighted.
3. The major draw-back of the study is lack of experimental validation of the genomic alterations and the functional significance of such alteration. It is discernible that the author reports an in silico analysis, however the relevance of the report warrants some sort of validation.
4. Though the lineage association has been analyzed for the inversions listed in Table-1, the lineage specific frequency for such inversions should have been mentioned. Were such inversions detected in all the representative assemblies analyzed for corresponding lineages?

Reviewer #3 (Comments for the Author):

The article by Nikhil Bhalla aims to identify inversions in the genome of Mycobacterium tuberculosis from both short-read and long-read sequencing data. Raw whole-genome sequencing data (long and short reads) were downloaded from public repositories. The author takes an unconventional approach in assembling short-read data de novo and using the assembled contigs as long reads to identify structural variations, particularly inversions. They show that lineage-specific inversions can be detected using assembled short reads. The manuscript addresses a specific question using computational methods and presents preliminary findings. It is not clear from the manuscript what effect these inversions have on the pathogen's biology and how these findings can be applied.

Major concerns:
1. How did the assembled short reads and long reads perform comparatively in detecting inversions? Were all structural variants called accurately in both approaches?
2. What mode and parameters were used when correcting assemblies using ragtag? If a reference sequence was used, which one?
3. Each axis on the cPC plot only explains ~7 and ~8% of the total variance seen in the data. Why?
Other concerns:
1. The manuscript can be edited, and some sections rewritten for better readability.
2. Throughout the manuscript, several abbreviations are used without first defining them.

3. The methods require more detail, especially parameters set for tools used in the study, will need to be mentioned if the findings are to be reproduced.
4. Wherever genomic coordinates are mentioned, please indicate the accession of the sequence for which the coordinates are relevant.

09.11.2025

Microbiology Spectrum

"Lineage-associated small inversions disrupt *dosT*, *dnaE2*, and a promoter-adjacent region in *Mycobacterium tuberculosis*.

Control no. Spectrum02469-25

Dear Author,

There is overall consistency throughout the manuscript. It is well designed. The discussion does not include references from (2025). It is novel and interesting, but there are several minor changes that should be considered to make it a perfect paper. Please consider the following comments:

1. There are so many abbreviations without full form, when you mention something, you have to write it in full form first and then write the abbreviations in the next paragraphs.
2. Regarding the conclusion, you just mentioned it in the abstract, there is no detailed conclusion for the manuscript.
3. How long you need to do this work?

With regards,

Fairuz Hassan Abdullah, Ph.D.

Biotechnology and Molecular Biology Lecturer

Point by point response to the comments asked by reviewers

We sincerely thank the Editor and the Reviewers for their time and effort to review our manuscript and providing us their valuable suggestions. We have revised the manuscript by incorporating the recommended modifications and additional information. Detailed point-by-point responses to all comments are provided below.

Reviewer's comments:

Reviewer #1: (Comments for the Author): 09.11.2025 Microbiology Spectrum "Lineage-associated small inversions disrupt dosT, dnaE2, and a promoter-adjacent region in Mycobacterium tuberculosis. Control no. Spectrum02469-25. Dear Author, There is overall consistency throughout the manuscript. It is well designed. The discussion does not include references from (2025). It is novel and interesting, but there are several minor changes that should be considered to make it a perfect paper.

Response: We are thankful to the Reviewer for the positive evaluation of our manuscript and for recognizing its overall consistency, sound design and novelty. In response to the Reviewer's suggestion, we have updated the "Discussion section" with additional text included in the revised manuscript, i.e. as follows:

Line 2 of discussion section, "n a recent study, a novel tool was developed that identified a structural variation in virulence-associated ESX-5 encoding gene. In addition, they also genotyped 41,134 isolates and found association of some SVs with resistance to various first and second-line drugs (29). "

New References: "Canalda-Baltrons A, Silcocks M, Hall MB, Theys D, Viberg LT, Sherry NL, Coin L, Dunstan SJ. Genome graphs reveal the importance of structural variation in Mycobacterium tuberculosis evolution and drug resistance. Nature Communications. 2025 Nov 28;16(1):10746".

Reviewer #1: There are so many abbreviations without full form, when you mention something, you have to write it in full form first and then write the abbreviations in the next paragraphs.

Response: We thank the Reviewer for this valuable and important suggestion. We have revised the entire manuscript to ensure that all abbreviations are defined at their first occurrence followed by the corresponding abbreviation in parentheses. This revision will significantly improve the clarity.

Reviewer #1: Regarding the conclusion, you just mentioned it in the abstract, there is no detailed conclusion for the manuscript.

Response: We appreciate the Reviewer's insightful comment regarding the incorporation of conclusion. In accordance with this suggestion, the manuscript has been revised to include a dedicated "Conclusion section".

"**Conclusion:** The current study provides analysis of genomic inversions in *Mycobacterium tuberculosis* and highlights their potential as an underappreciated source of genetic evolution

and lineage-specific phenotypic diversification. This study establishes a framework for the identification and validation of lineage-associated inversion events, by integrating *de novo* assembly-based analyses of short-read sequencing data with independent long-read sequencing and structural variant detection. From the analysis we speculate that that the identified inversions may influence gene expression by altering regulatory regions, disrupting coding sequences, facilitating the formation of fusion genes, and introducing nonsense mutations that can potentially affect overall gene function. Furthermore, the yet-to-be-identified inversions in the *M. tuberculosis* genome may affect genes involved in stress adaptation, DNA repair, metabolism, and regulatory processes."

Reviewer #1: How long you need to do this work?

Response: We thank the Reviewer for this important question. The entire study was conducted over a period of approximately one year**,** including study design, data generation, quality assessment, bioinformatics analyses, interpretation of the results, and manuscript preparation.

We again thank Reviewer 1 for their feedback and we trust that these revisions will substantially improve quality of the manuscript.

Responses to the comments by Reviewer 2 as follows:

Reviewer #2: (Comments for the Author): In the article "Lineage-associated small inversions disrupt dosT, dnaE2, and a promoter-adjacent region in *Mycobacterium tuberculosis*", Bhalla N reports small inversions with possible impact in gene structure, expression linked to lineage specific phenotypic attributes. The report is intensive in identifying small inversions in various lineages. The methodologies are well implemented and well-drafted. The statistical analysis is also robust. The most elegant aspect of the study is analyzing short read and long read assemblies.

Response: We sincerely thank the Reviewer for evaluating the manuscript thoroughly and for acknowledging the robustness of the analytical framework, the methodological approach and for highlighting the strategy employed in the study. The Reviewer's positive assessment is highly encouraging and motivates us to continue improving the quality and impact of our work.

Reviewer #2: The PCA for short and long read assembly data should be elaborated.

Response: We appreciate the Reviewer's insightful suggestion. In the revised manuscript, we have modified the "Method section", including description of the Principal Coordinate Analysis (PCA) performed on both short-read and long-read assembly datasets.

The modified Methods section is included in the revised manuscript:

"The alignment backbone output was parsed to identify LCBs, and inversion-associated LCB orientation values were converted into binary variables, where positive and negative orientations were coded as 1 and 0, respectively. The resulting binary inversion matrix was subjected to categorical Principal Coordinate Analysis (PCA), using the FactoMineR R

package (22). The first two Categorical Principal coordinate (cPC) axes were plotted to visualize lineage-associated clustering of inversion-derived structural variation patterns".

Reviewer #2: The significance of the study should have been highlighted.

Response: We thank the Reviewer for this valuable suggestion, also given by reviewer 1. As mentioned earlier in response, we have added the conclusion in the manuscript.

Reviewer #2: The major draw-back of the study is lack of experimental validation of the genomic alterations and the functional significance of such alteration. It is discernible that the author reports an in silico analysis, however the relevance of the report warrants some sort of validation.

Response: We thank the Reviewer for this criticism. We acknowledge that experimental validation of the identified genomic inversions and their functional consequences would have added value to our findings. We have added this as a limitation of the study in "Discussion" in the revised manuscript.

The additional text included in Discussion section in the revised manuscript:

"Moreover, future experimental validation studies are required to confirm the identified inversions and elucidate their precise contribution to *Mycobacterium tuberculosis* physiology, adaptation, virulence, and lineage-specific evolution. The primary objective of the study was to perform a pilot scale genome-wide identification of lineage-associated small inversions (if any) using complementary short-read and long-read sequencing datasets. Functional validation through approaches such as polymerase chain reaction (PCR)-based confirmation, transcriptomic analyses, gene expression studies, targeted mutagenesis, and phenotypic characterization may be carried out by researchers having access to isolates and necessary facilities. We believe that, despite the absence of experimental validation, the systematic identification of lineage-associated small inversions using independent sequencing strategies provides an important information pertaining to the evolution of this deadly bacteria."

Reviewer #2: Though the lineage association has been analyzed for the inversions listed in Table-1, the lineage specific frequency for such inversions should have been mentioned. Were such inversions detected in all the representative assemblies analyzed for corresponding lineages?

Response: We appreciate the Reviewer for this feedback. We have incorporated the additional changes in Table 1 in revised manuscript, which includes lineage-specific frequency of each statistically significant associated inversion. For information, the identified inversions are not found in in all representative assemblies of the corresponding lineages. Instead, they showed some degree of lineage-associated enrichment based on Fisher's exact test.

The modified "Table 1" is included in the revised manuscript:

Table 1: Summary of statistically significant inversions. Inversions encompassed CDS and control elements disrupting or potentially altering their expression, respectively. The complete *Mycobacterium tuberculosis* reference genome (RefSeq: NC_000962.3) was used P values were determined using Fisher's exact test to analyse their lineage associations. Start and End give the inversion breakpoint coordinates in base pairs relative to the *M. tuberculosis H37Rv*

reference genome (RefSeq: NC_000962.3), and Genomic region indicates the overlapping gene or control element with its Rv locus tag. For each inversion, Carriers in lineage and Carriers in others report the number of isolates carrying the inversion out of the total isolates analysed within the indicated lineage and across all other lineages combined, respectively, while Overall carriers gives the corresponding count for the full dataset; Freq in lineage (%), Freq in others (%), and Overall freq (%) express these counts as the respective percentages. Association denotes the direction of the lineage relationship, with Positive indicating enrichment and Negative indicating depletion of the inversion within the indicated lineage. Abbreviations: S. No.: serial number; CDS: coding sequence; Freq: Frequency; Raw P: uncorrected Fisher's exact test p-value; BH: Benjamini–Hochberg adjusted p-value (false discovery rate).

S. No.	Lineage	Start	End	Raw P	BH P-value	Genomic region	Carriers in lineage	Freq in lineage (%)	Carriers in others	Freq in others (%)	Overall carriers	Overall freq (%)	Association
1	Lineage 1	76531	77183	1.52E-04	1.69E-02	sdaA (Rv0069c)	8/9	88.9	56/226	24.8	64/235	27.2	Positive
2	Lineage 1	1482828	1483138	6.68E-03	8.03E-02	Rv1320c	6/9	66.7	50/226	22.1	56/235	23.8	Positive
3	Lineage 3	1508080	1508719	1.54E-02	1.40E-01	Rv1341, Rv1342c, lprD (Rv1343c)	3/6	50	21/229	9.2	24/235	10.2	Positive
4	Lineage 3	2272338	2273019	4.09E-02	1.87E-01	Rv2026c, dosT (Rv2027c)	3/6	50	31/229	13.5	34/235	14.5	Positive
5	Lineage 3	3781352	3781891	5.38E-03	9.92E-02	Rv3369, dnaE2 (Rv3370c)	5/6	83.3	57/229	24.9	62/235	26.4	Positive
6	Lineage 4	869783	869980	2.79E-02	2.29E-01	CDS flanked by Rv0776c (upstream) and purB (Rv0777) (downstream)	8/112	7.1	21/123	17.1	29/235	12.3	Negative
7	Lineage 4	1508080	1508719	8.46E-03	1.19E-01	Rv1341, Rv1342c, lprD (Rv1343c)	5/112	4.5	19/123	15.4	24/235	10.2	Negative
8	Lineage 4	2844929	2845174	1.03E-03	2.91E-02	fas (Rv2524c)	15/112	13.4	39/123	31.7	54/235	23	Negative
9	Lineage 4	3781352	3781891	2.71E-02	2.26E-01	Rv3369, dnaE2 (Rv3370c)	22/112	19.6	40/123	32.5	62/235	26.4	Negative

Reviewer #3: (Comments for the Author): The article by Nikhil Bhalla aims to identify inversions in the genome of *Mycobacterium tuberculosis* from both short-read and long-read sequencing data. Raw whole-genome sequencing data (long and short reads) were downloaded from public repositories. The author takes an unconventional approach in assembling short-read data de novo and using the assembled contigs as long reads to identify structural variations, particularly inversions. They show that lineage-specific inversions can be detected using assembled short reads. The manuscript addresses a specific question using computational methods and presents preliminary findings. It is not clear from the manuscript what effect these inversions have on the pathogen's biology and how these findings can be applied.

Response: We thank the Reviewer for this valuable suggestion, also given by reviewer 1 and 2. As mentioned earlier in response, we have added the conclusion in the manuscript. .

Also, we completely agree with this point that, the present study is primarily computational and further its functional consequences of the identified inversions remain to be experimentally validated. Accordingly, we have included this as a limitation of the study in "Discussion section" in revised manuscript, as already explained above.

Reviewer #3: How did the assembled short reads and long reads perform comparatively in detecting inversions? Were all structural variants called accurately in both approaches?

Response: We thank the Reviewer for this important question. The assembled short-read approach provided preliminary signals of candidate inversions and lineage-associated clustering, however, it was not considered sufficient for definitive structural variant detection because short-read assemblies are limited by contig fragmentation and reduced resolution of complex rearrangements. In contrast, long-read sequencing provided more reliable inversion detection because long reads can span larger genomic regions and resolve structural rearrangement breakpoints more accurately. Therefore, not all structural variants were detected concordantly or with equal confidence by both approaches. The long-read-derived calls were considered high-confidence, whereas the assembled short-read analysis was used as a supportive exploratory approach.

Reviewer #3: What mode and parameters were used when correcting assemblies using ragtag? If a reference sequence was used, which one?

Response: We sincerely thank the Reviewer's request for clarification regarding the RagTag-based assembly correction step. This information has been incorporated in "Methods section" in revised manuscript for reproducibility of the data.

The additional text included in "Method section", in the revised manuscript:

"The consistency of assemblies were improved using ragtag prior to downstream structural variation analysis. The complete *Mycobacterium tuberculosis* H37Rv genome (**RefSeq accession NC_000962.3)** was used as the reference genome. RagTag was run in **correction mode** using the "`ragtag.py correct`" command with default parameters. This correction step was included to reduce the effect of potential assembly inconsistencies before generating Locally Collinear Block (LCB) based inversion and translocation matrices".

Reviewer #3: Each axis on the cPC plot only explains ~7 and ~8% of the total variance seen in the data. Why?

Response: We appreciate the Reviewer's observation. In this study, Principal coordinate total variance percentage were low because the inversions alone are not sufficient to explain all variance, since the analysis was performed on a high-dimensional binary LCB-derived matrix, where inversion patterns were converted into categorical 0/1 variables. In such datasets, the total variation is typically distributed across multiple structural features rather than being captured by only one or two dominant components. This suggests that the lineage-specific inversion signal is present but distributed across several LCB features.

Reviewer #3: The manuscript can be edited, and some sections rewritten for better readability.

Response: We thank Reviewer's valuable suggestion. In response, the manuscript has been significantly revised by incorporating changes or additional information in methodology, discussion and conclusion sections.

Reviewer #3: Throughout the manuscript, several abbreviations are used without first defining them.

Response: We acknowledge the Reviewer's careful observation. In response, the manuscript has been thoroughly revised to ensure that all abbreviations are defined at their first occurrence followed by the corresponding abbreviation in parentheses.

Reviewer #3: The methods require more detail, especially parameters set for tools used in the study, will need to be mentioned if the findings are to be reproduced.

Response: We appreciate the Reviewer's important suggestion regarding methodological reproducibility. In response, the Methods section has been revised in details. Specifically, we have clarified the steps involved ragtag and PCA for short-read assembly and long-read assembly to enable reproducibility of the findings. These revisions will provide more transparency and should allow readers to understand better and reproduce the computational approach used in the study.

The modified Methods section is included in the revised manuscript:

"Detecting Inversions using assemblies made using short-read sequencing: The short reads were first adapter-trimmed using bbduk.sh. Adapter-trimmed reads were subjected to Shovill (v1.1.0) (assembler: megahit) for *de novo* assembly and polishing (17,18). The consistency of assemblies were improved using ragtag prior to downstream structural variation analysis. The complete *Mycobacterium tuberculosis* H37Rv genome (**RefSeq accession NC_000962.3)** was used as the reference genome. RagTag was run in correction mode using the "ragtag.py correct" command. This correction step was included to reduce the effect of potential assembly inconsistencies before generating Locally Collinear Block (LCB) based inversion and translocation matrices. The corrected assemblies generated by RagTag were subsequently subjected to WGS-based drug resistance and lineage detection using tb-profiler (v6.6.3) (19). The assemblies were subjected to ParsNP (v1.2) for the determination of distances (20). The tree from parsnp, the interactive tree of life (itol) files from the tb-profiler output, were used to generate an annotated dendrogram. A phylogenetic free distance matrix was created, and 10 replicates per lineage were selected for analysis with ProgressiveMauve (v20150226) with and without the assumption of collinearity (21). Apart from 10 replicates per lineage, a representative complete *Mycobacterium tuberculosis* H37Rv genome (RefSeq: NC_000962.3) was also included in the Progressive Mauve alignment. The alignment backbone output was parsed to identify LCBs, and inversion-associated LCB orientation values were converted into binary variables, where positive and negative orientations were coded as 1 and 0, respectively. The resulting binary inversion matrix was subjected to categorical Principal Coordinate Analysis (PCA), using the FactoMineR R package (22). The first two Categorical Principal

coordinate (cPC) axes were plotted to visualize lineage-associated clustering of inversion-derived structural variation patterns".

Reviewer #3: Wherever genomic coordinates are mentioned, please indicate the accession of the sequence for which the coordinates are relevant.

Response: We thank the Reviewer's important suggestion. In response, the manuscript has been revised to specify the corresponding sequence accession wherever genomic coordinates are reported including the manuscript, tables, and figure legends.

We trust that the revisions implemented in response to the Reviewer's comments will strengthen the manuscript by improving its clarity. We sincerely appreciate the Reviewer's once again for their valuable suggestions.

Re: Spectrum02469-25R1 (Lineage-associated small inversions disrupt *dosT*, *dnaE2*, and a promoter-adjacent region in some *Mycobacterium tuberculosis isolates*.)

Dear Dr. Nikhil Bhalla:

Your manuscript has been accepted, and I am forwarding it to the ASM production staff for publication. Your paper will first be checked to make sure all elements meet the technical requirements. ASM staff will contact you if anything needs to be revised before copyediting and production can begin. Otherwise, you will be notified when your proofs are ready to be viewed.

Data Availability: ASM policy requires that data be available to the public upon online posting of the article, so please verify all links to sequence records, if present, and make sure that each number retrieves the full record of the data. If a new accession number is not linked or a link is broken, provide production staff with the correct URL for the record. If the accession numbers for new data are not publicly accessible before the expected online posting of the article, publication may be delayed; please contact ASM production staff immediately with the expected release date.

Publication Fees: For information on publication fees and which article types have charges, please visit ourwebsite. We have partnered with Copyright Clearance Center (CCC) to collect author charges. If fees apply to your paper, you will receive a message from no-reply@copyright.com with further instructions. For questions related to paying charges through RightsLink, please contact CCC at ASM_Support@copyright.com or toll free at +1-877-622-5543. CCC makes every attempt to respond to all emails within 24 hours.

ASM Membership: Corresponding authors may join or renew ASM membership to obtain discounts on publication fees. Need to upgrade your membership level? Please contact Customer Service at Service@asmusa.org.

Embargo Policy: A press release may be issued as soon as the manuscript is posted on theSpectrum Latest Articles webpage. The corresponding author will receive an email with the subject line "ASM Journals Author Services Notification" when the article is available online.

Cover Image Submissions: If you would like to submit a potential Cover Image, please email a file and a short legend to spectrum@asmusa.org. Please note that we can only consider images that (i) the authors created or own and (ii) have not been previously published. By submitting, you agree that the image can be used under the same terms as the published article. Image File requirements: TIF/EPS, 7.5 inches wide by 8.25 inches tall (at least 2,250 pixels wide by 2,475 pixels tall), minimum 300 dpi resolution (600 dpi preferred), RGB, and no figure elements, e.g., arrows or panel labels. The legend should be a short description of the image, 1-2 sentences recommended. Please download and use this interactive template in Adobe the https://journals.asm.org/pb-assets/html/asm-cover-sizing-tool.html to ensure that your proposed cover image meets our size requirements (https://journals.asm.org/pb-assets/pdf-text-excel-files/ASM-Interactive-Sizing-Cover-Template-1715689791.pdf).

The ASM Journals program strives for constant improvement in our submission and publication process. Please tell us how we can improve your experience by taking this quick Author Survey.

Thank you for submitting your paper to Spectrum.

Sincerely,
Felix Toka
Editor
Microbiology Spectrum

Reviewer #1 (Comments for the Author):

Dear Author,

Thank you for your comprehensive and detailed responses to every comment made in the first review. The thorough revisions you've made reflect your earnest efforts to rectify the issues raised and enhance the overall quality of the manuscript. The recent literature cited in 2025 has been added to the work, complete definition of abbreviations and introduction of a dedicated Conclusion section has greatly enhanced the work. Thanks for the detail and clarity on each change and exact location of each change.

Reviewer #2 (Comments for the Author):

The authors habe addressed the points raised earlier and incorporated necesary modifucations.

Comment:
The authors have mentioned that the accession numbers in a supplementary table, but all the numbers should be mentioned in core-text data availabiluty segment.
