# Supplementary material for: Lineage-associated small inversions disrupt dosT, dnaE2, and a promoter-adjacent region in some Mycobacterium tuberculosis isolates
Source: Microbiol Spectr. 2026 Aug 10;14(9):e02469-25. doi: 10.1128/spectrum.02469-25 (PMC13532295; doi:10.1128/spectrum.02469-25)
Supplement: Supplementary file 2 [file spectrum.02469-25-s0001.docx]

**Title:** Lineage-associated small inversions disrupt *dosT*, *dnaE2*, and a promoter-adjacent region in some *Mycobacterium tuberculosis* isolates.

**Supplementary Figures and Tables**

**Figure S1: Genetic inversions in mixed lineage datasets also showed lineage-specific associations found in pure lineages.** A: No inversion with a breakpoint on both strands showed a significant association with Lineage 2. B: In the dataset containing reads from mixed lineages, Lineages 2 and 4 showed some associations that were also found in datasets of pure lineage 4 samples. Abbreviations: INV: Inversions; BND: BreakEND; nonsig: non-significant.

**Table S1:** Short and Long read sequencing read Bioprojects, data sets per bioproject (n) and lineage distribution. All data sets per bioproject could not be assembled for lineage profiling. Therefore value of n may be more than number of samples in each lineage from that BioProject. Other consisted of datasets with mixed lineages. *La1 samples. Randomized sample size was 500 and after deduplication was reduced further.

| **S. No.** | **BioProject IDs** | **Lineage 1** | **Lineage 2** | **Lineage 3** | **Lineage 4** | **Other** | **Sequencing type** |
| --- | --- | --- | --- | --- | --- | --- | --- |
| 1 | PRJEB8783 (n=54) | 4 | 2 | 4 | 28 | 9 | Long read |
| 2 | PRJEB73759 (n=34) | - | - | - | 25 | - | Long read |
| 3 | PRJNA586859 (n=24) | - | 2 | - | 22 | - | Long read |
| 4 | PRJEB29197 (n=15) | 3 | - | - | 8 | 2 | Long read |
| 5 | PRJNA1019874 (n=14) | - | 12 | - | 2 | - | Long read |
| 6 | PRJNA719670 (n=13) | - | 1 | - | 12 | - | Long read |
| 7 | PRJEB23996 (n=12) | - | - | - | 8 | - | Long read |
| 8 | PRJNA1021585 (n=9) | - | 2 | - | 1 | 5 | Long read |
| 9 | PRJNA1023168 (n=9) | - | 2 | - | 1 | 6 | Long read |
| 10 | PRJNA436997 (n=7) | - | - | - | 2 | - | Long read |
| 11 | PRJNA480888 (n=6) | - | - | - | - | - | Long read |
| 12 | PRJNA231221 (n=6) | 1 | - | 2 | - | - | Long read |
| 13 | PRJNA236012 (n=5) | - | - | - | - | - | Long read |
| 14 | PRJNA587747 (n=5) | - | 2 | - | 3 | - | Long read |
| 15 | PRJNA428763 (n=5) | - | - | - | - | 5* | Long read |
| 16 | PRJEB57950 (n=4) | - | - | - | - | - | Long read |
| 17 | PRJNA573545 (n=3) | - | - | - | - | - | Long read |
| 18 | PRJNA505382 (n=3) | - | 3 | - | - | - | Long read |
| 19 | PRJNA421446 (n=2) | - | - | - | - | - | Long read |
| 20 | PRJNA813637 (n=2) | - | - | - | - | - | Long read |
| 21 | PRJNA556438 (n=1) | - | - | - | - | - | Long read |
| 22 | PRJNA598991 (n=1) | - | - | - | - | - | Long read |
| 23 | PRJNA578162 (n=1) | - | - | - | 1 | - | Long read |
| 24 | PRJNA833906 (n=1) | 1 | - | - | - | - | Long read |
| 25 | PRJNA549270 (n=1) | - | - | - | - | - | Long read |
| 26 | PRJEB41116 (n=randomized) | 63 | 163 | 157 | 61 | 4 | Short read |
| 27 | PRJNA879962 (n=randomized) | 1 | 15 | 0 | 3 | 0 | Short read |

**Table S2: Assembly details:** The WGS long reads were assembled using Raven and subsequently analysed using Quast. The Assemblies marked in bold were eliminated due to poor quality from subsequent analysis. Some other assemblies were also removed because of mixed lineage reads per assembly.

| **S. No.** | **SRA accession ID** | **GC (%)** | **Genome fraction (%)** | **N50** | **Total length** | **Misassemblies** |
| --- | --- | --- | --- | --- | --- | --- |
| **1** | **ERR968284** | **61.46** | **0.353** | **15638** | **15638** | **0** |
| **2** | **ERR3273780** | **64.66** | **1.937** | **14734** | **86114** | **4** |
| **3** | **ERR964416** | **64.42** | **3.552** | **12487** | **160927** | **0** |
| **4** | **ERR3427690** | **61.02** | **4.893** | **21301** | **3934919** | **2** |
| **5** | **ERR964420** | **65.09** | **4.972** | **14790** | **227108** | **0** |
| **6** | **ERR968286** | **64.9** | **5.598** | **15129** | **254612** | **4** |
| **7** | **ERR964412** | **65.89** | **8.321** | **17448** | **382994** | **3** |
| **8** | **ERR964419** | **64.83** | **12.493** | **18240** | **563309** | **9** |
| **9** | **ERR956957** | **64.59** | **12.967** | **22299** | **582950** | **8** |
| **10** | **ERR3273781** | **65.13** | **14.219** | **16692** | **632899** | **5** |
| **11** | **ERR964414** | **65.81** | **24.636** | **21081** | **1119116** | **16** |
| **12** | **ERR3427707** | **65.43** | **29.395** | **15845** | **1307442** | **4** |
| 13 | ERR3253133 | 65.37 | 39.541 | 19109 | 1755932 | 11 |
| 14 | ERR968285 | 65.63 | 44.396 | 24662 | 1993204 | 22 |
| 15 | SRR26193462 | 65.52 | 63.185 | 21739 | 2809354 | 40 |
| 16 | SRR26252773 | 65.52 | 63.185 | 21739 | 2809354 | 40 |
| 17 | SRR26252766 | 65.5 | 67.224 | 22181 | 2988763 | 57 |
| 18 | ERR3253134 | 65.49 | 67.762 | 29517 | 3005787 | 20 |
| 19 | ERR964407 | 65.52 | 72.542 | 41492 | 3256540 | 38 |
| 20 | ERR3427701 | 65.4 | 73.299 | 29845 | 3286443 | 23 |
| 21 | ERR964410 | 65.49 | 73.532 | 41480 | 3306035 | 46 |
| 22 | ERR964405 | 65.47 | 75.5 | 42758 | 3394386 | 40 |
| 23 | ERR904398 | 65.54 | 76.531 | 36302 | 3388300 | 2 |
| 24 | ERR964421 | 65.53 | 77.851 | 44277 | 3502337 | 40 |
| 25 | ERR3427702 | 65.43 | 78.486 | 32856 | 3528605 | 22 |
| 26 | ERR956959 | 65.6 | 79.536 | 44550 | 3561293 | 34 |
| 27 | ERR3427699 | 65.48 | 81.168 | 38431 | 3653545 | 39 |
| 28 | SRR6467892 | 65.63 | 86.443 | 42060 | 4308563 | 127 |
| 29 | ERR1025289 | 65.51 | 86.78 | 73257 | 3879049 | 37 |
| 30 | ERR904406 | 65.55 | 87.659 | 64214 | 3882226 | 2 |
| 31 | ERR964409 | 65.45 | 89.08 | 65803 | 3983433 | 33 |
| 32 | ERR956960 | 65.64 | 89.341 | 72845 | 4038446 | 48 |
| 33 | ERR3427693 | 65.51 | 89.461 | 53338 | 3979860 | 20 |
| 34 | ERR964406 | 65.6 | 89.588 | 74040 | 4031773 | 43 |
| 35 | ERR964417 | 65.56 | 89.743 | 66408 | 4017987 | 40 |
| 36 | ERR3427705 | 65.5 | 89.986 | 87237 | 4044257 | 29 |
| 37 | SRR26193459 | 65.65 | 90.284 | 121336 | 4016698 | 63 |
| 38 | SRR26252770 | 65.65 | 90.284 | 121336 | 4016698 | 63 |
| 39 | ERR3253136 | 65.45 | 90.299 | 52283 | 4019100 | 39 |
| 40 | ERR3273782 | 65.52 | 90.557 | 73300 | 4034342 | 41 |
| 41 | SRR26193457 | 65.67 | 91.467 | 151146 | 4071612 | 67 |
| 42 | SRR26252768 | 65.67 | 91.467 | 151146 | 4071612 | 67 |
| 43 | ERR904401 | 65.51 | 92.222 | 75354 | 4080445 | 4 |
| 44 | SRR26193461 | 65.68 | 92.558 | 148529 | 4142882 | 72 |
| 45 | SRR26252772 | 65.68 | 92.558 | 148529 | 4142882 | 72 |
| 46 | SRR26193460 | 65.63 | 93.456 | 124756 | 4155244 | 52 |
| 47 | SRR26252771 | 65.63 | 93.456 | 124756 | 4155244 | 52 |
| 48 | ERR3427703 | 65.57 | 93.564 | 87945 | 4167202 | 33 |
| 49 | ERR904397 | 65.59 | 94.609 | 123088 | 4198096 | 5 |
| 50 | ERR904410 | 65.55 | 94.636 | 90395 | 4192128 | 3 |
| 51 | SRR6467886 | 65.55 | 95.275 | 83989 | 4814596 | 123 |
| 52 | ERR3427700 | 65.58 | 95.566 | 149153 | 4249379 | 18 |
| 53 | ERR904399 | 65.59 | 95.621 | 110682 | 4246256 | 4 |
| 54 | ERR904414 | 65.52 | 96.117 | 134004 | 4253601 | 4 |
| 55 | ERR904415 | 65.58 | 96.351 | 130503 | 4271272 | 3 |
| 56 | ERR904413 | 65.58 | 96.434 | 180886 | 4270236 | 5 |
| 57 | SRR26139272 | 65.6 | 96.644 | 356832 | 4331922 | 57 |
| 58 | ERR964418 | 65.57 | 96.661 | 423386 | 4306116 | 45 |
| 59 | ERR3253135 | 65.59 | 96.825 | 158500 | 4309958 | 44 |
| 60 | ERR3427692 | 65.55 | 97 | 159022 | 4342330 | 30 |
| 61 | SRR26193458 | 65.58 | 97.052 | 1770297 | 4305459 | 41 |
| 62 | SRR26252769 | 65.58 | 97.052 | 1770297 | 4305459 | 41 |
| 63 | ERR3427704 | 65.55 | 97.08 | 148504 | 4353849 | 27 |
| 64 | SRR26193456 | 65.62 | 97.514 | 3194304 | 4329496 | 58 |
| 65 | SRR26252767 | 65.62 | 97.514 | 3194304 | 4329496 | 58 |
| 66 | ERR12742682 | 65.64 | 97.522 | 478188 | 4340775 | 42 |
| 67 | ERR12743217 | 65.64 | 97.522 | 478188 | 4340775 | 42 |
| 68 | ERR964411 | 65.6 | 97.561 | 246319 | 4348805 | 47 |
| 69 | SRR6467888 | 65.55 | 97.66 | 265986 | 4822807 | 76 |
| 70 | SRR6467884 | 65.63 | 97.724 | 1060367 | 4419853 | 37 |
| 71 | ERR964401 | 65.6 | 97.74 | 376215 | 4377948 | 44 |
| 72 | SRR6467890 | 65.63 | 97.795 | 3383998 | 4357154 | 31 |
| 73 | ERR956955 | 65.6 | 97.875 | 394172 | 4383072 | 46 |
| 74 | SRR26193447 | 65.63 | 98.171 | 1513760 | 4380848 | 44 |
| 75 | SRR26252774 | 65.63 | 98.171 | 1513760 | 4380848 | 44 |
| 76 | SRR26139270 | 65.6 | 98.18 | 2394752 | 4377572 | 46 |
| 77 | SRR26282616 | 65.61 | 98.22 | 4357721 | 4357721 | 43 |
| 78 | SRR10828834_subreads | 65.62 | 98.243 | 4380173 | 4380173 | 60 |
| 79 | ERR956958 | 65.59 | 98.248 | 427507 | 4402800 | 56 |
| 80 | SRR10520596_subreads | 65.63 | 98.279 | 3310173 | 4386921 | 35 |
| 81 | SRR26139249 | 65.61 | 98.349 | 4381333 | 4381333 | 44 |
| 82 | ERR964402 | 65.6 | 98.402 | 3493041 | 4420654 | 28 |
| 83 | SRR26139250 | 65.61 | 98.449 | 4379842 | 4379842 | 43 |
| 84 | ERR904416 | 65.62 | 98.48 | 257317 | 4386384 | 2 |
| 85 | SRR26282622 | 65.61 | 98.535 | 3308009 | 4403718 | 38 |
| 86 | SRR26283526 | 65.6 | 98.668 | 4395605 | 4395605 | 53 |
| 87 | ERR3427691 | 65.63 | 98.698 | 305932 | 4426031 | 32 |
| 88 | ERR956956 | 65.6 | 98.738 | 1067527 | 4441428 | 30 |
| 89 | SRR26139273 | 65.6 | 98.758 | 4400816 | 4400816 | 53 |
| 90 | SRR17231461_subreads | 65.65 | 98.792 | 4397364 | 4404546 | 42 |
| 91 | SRR26139269 | 65.62 | 98.793 | 4396382 | 4396382 | 45 |
| 92 | SRR26139253 | 65.6 | 98.796 | 4403280 | 4403280 | 53 |
| 93 | ERR12743218 | 65.59 | 98.808 | 4394135 | 4394135 | 42 |
| 94 | SRR26139248 | 65.61 | 98.813 | 4399469 | 4399469 | 48 |
| 95 | SRR26282628 | 65.62 | 98.837 | 2365089 | 4419976 | 40 |
| 96 | ERR3253132 | 65.59 | 98.843 | 564688 | 4394374 | 45 |
| 97 | SRR26282620 | 65.62 | 98.867 | 2885526 | 4416486 | 42 |
| 98 | SRR28788881 | 65.6 | 98.88 | 4420838 | 4420838 | 34 |
| 99 | SRR26282624 | 65.61 | 98.888 | 1550541 | 4414982 | 42 |
| 100 | SRR26282615 | 65.61 | 98.903 | 3328888 | 4413794 | 52 |
| 101 | SRR26139247 | 65.61 | 98.91 | 4415164 | 4415164 | 46 |
| 102 | SRR26282627 | 65.64 | 98.911 | 4396945 | 4412519 | 52 |
| 103 | SRR8186770 | 65.6 | 98.913 | 4411592 | 4411592 | 57 |
| 104 | SRR26282634 | 65.62 | 98.916 | 4393939 | 4393939 | 36 |
| 105 | SRR26139274 | 65.62 | 98.918 | 4409966 | 4409966 | 52 |
| 106 | SRR26283527 | 65.6 | 98.921 | 4409966 | 4409966 | 54 |
| 107 | ERR12743215 | 65.6 | 98.935 | 3267386 | 4412090 | 43 |
| 108 | SRR8186771 | 65.6 | 99.001 | 4421780 | 4421780 | 57 |
| 109 | ERR12743225 | 65.6 | 99.007 | 4395757 | 4395757 | 41 |
| 110 | ERR12743219 | 65.58 | 99.011 | 4392073 | 4392073 | 42 |
| 111 | SRR26282612 | 65.64 | 99.018 | 4399765 | 4399765 | 45 |
| 112 | SRR17234896 | 65.59 | 99.02 | 4423688 | 4423688 | 56 |
| 113 | SRR26283524 | 65.62 | 99.026 | 3553085 | 4412983 | 42 |
| 114 | ERR10558090 | 65.66 | 99.039 | 4426559 | 4426559 | 40 |
| 115 | SRR26139262 | 65.61 | 99.049 | 4414432 | 4414432 | 53 |
| 116 | ERR12743222 | 65.6 | 99.052 | 1003438 | 4409422 | 41 |
| 117 | SRR26139271 | 65.61 | 99.057 | 4412699 | 4412699 | 55 |
| 118 | SRR8186772 | 65.6 | 99.059 | 4422477 | 4422477 | 57 |
| 119 | ERR12742692 | 65.6 | 99.065 | 4398419 | 4398419 | 42 |
| 120 | ERR12743227 | 65.6 | 99.065 | 4398419 | 4398419 | 42 |
| 121 | ERR964415 | 65.6 | 99.065 | 550492 | 4422635 | 47 |
| 122 | SRR17231409 | 65.64 | 99.072 | 4349594 | 4461376 | 47 |
| 123 | SRR26282630 | 65.61 | 99.084 | 4416312 | 4416312 | 51 |
| 124 | SRR26282633 | 65.61 | 99.085 | 4397498 | 4397498 | 29 |
| 125 | SRR20998236_subreads | 65.58 | 99.099 | 1006032 | 4470854 | 52 |
| 126 | SRR17234887 | 65.64 | 99.101 | 2781550 | 4433386 | 42 |
| 127 | SRR17231612_subreads | 65.63 | 99.103 | 4439789 | 4439789 | 59 |
| 128 | SRR17234897 | 65.61 | 99.124 | 3337695 | 4429204 | 38 |
| 129 | SRR26282611 | 65.62 | 99.144 | 4395662 | 4395662 | 32 |
| 130 | SRR17231613_subreads | 65.64 | 99.156 | 4424083 | 4436074 | 29 |
| 131 | SRR17234886 | 65.61 | 99.161 | 1418193 | 4419730 | 32 |
| 132 | SRR20998128_subreads | 65.6 | 99.164 | 3898996 | 4447884 | 47 |
| 133 | ERR12743224 | 65.59 | 99.172 | 4401648 | 4401648 | 42 |
| 134 | ERR12742685 | 65.59 | 99.174 | 4401377 | 4401377 | 43 |
| 135 | ERR12743220 | 65.59 | 99.174 | 4401377 | 4401377 | 43 |
| 136 | SRR17231608 | 65.69 | 99.194 | 4434668 | 4434668 | 48 |
| 137 | ERR12742694 | 65.61 | 99.196 | 4412043 | 4412043 | 42 |
| 138 | ERR12743229 | 65.61 | 99.196 | 4412043 | 4412043 | 42 |
| 139 | SRR26283528 | 65.65 | 99.2 | 2356538 | 4414367 | 29 |
| 140 | ERR12743216 | 65.58 | 99.207 | 4407153 | 4407153 | 44 |
| 141 | ERR12743214 | 65.64 | 99.208 | 3744648 | 4418858 | 37 |
| 142 | ERR12742691 | 65.61 | 99.222 | 2904210 | 4419932 | 41 |
| 143 | ERR12743226 | 65.61 | 99.222 | 2904210 | 4419932 | 41 |
| 144 | SRR26283525 | 65.66 | 99.225 | 2465678 | 4436372 | 39 |
| 145 | SRR26282618 | 65.68 | 99.225 | 2352551 | 4442527 | 48 |
| 146 | ERR12743221 | 65.6 | 99.235 | 4402059 | 4402059 | 42 |
| 147 | SRR17234889 | 65.62 | 99.237 | 770418 | 4470956 | 42 |
| 148 | SRR26282614 | 65.61 | 99.248 | 2853918 | 4437682 | 36 |
| 149 | ERR12742695 | 65.59 | 99.256 | 4403601 | 4403601 | 40 |
| 150 | ERR12743230 | 65.59 | 99.256 | 4403601 | 4403601 | 40 |
| 151 | SRR26282626 | 65.61 | 99.259 | 4236102 | 4441520 | 53 |
| 152 | ERR964408 | 65.6 | 99.279 | 2225522 | 4427465 | 48 |
| 153 | ERR12742693 | 65.59 | 99.287 | 4405373 | 4405373 | 41 |
| 154 | ERR12743228 | 65.59 | 99.287 | 4405373 | 4405373 | 41 |
| 155 | SRR9176615 | 65.61 | 99.29 | 4438744 | 4438744 | 51 |
| 156 | SRR26282621 | 65.62 | 99.29 | 4240249 | 4415960 | 39 |
| 157 | ERR12742688 | 65.59 | 99.296 | 4404447 | 4404447 | 41 |
| 158 | ERR12743223 | 65.59 | 99.296 | 4404447 | 4404447 | 41 |
| 159 | ERR904417 | 65.6 | 99.3 | 582204 | 4417489 | 7 |
| 160 | SRR26282623 | 65.63 | 99.308 | 3968861 | 4435096 | 43 |
| 161 | SRR17234893 | 65.62 | 99.309 | 4431215 | 4431215 | 50 |
| 162 | SRR26282629 | 65.61 | 99.313 | 2430574 | 4433038 | 38 |
| 163 | SRR9588640_subreads | 65.61 | 99.313 | 4428686 | 4428686 | 50 |
| 164 | SRR26283454 | 65.64 | 99.322 | 4414792 | 4414792 | 38 |
| 165 | SRR17234894 | 65.61 | 99.323 | 4425018 | 4425018 | 47 |
| 166 | SRR26282617 | 65.61 | 99.329 | 4410090 | 4410090 | 32 |
| 167 | SRR26282619 | 65.57 | 99.339 | 4439910 | 4439910 | 52 |
| 168 | SRR17234891 | 65.64 | 99.34 | 2758798 | 4458414 | 50 |
| 169 | SRR17234900 | 65.59 | 99.341 | 4403788 | 4403788 | 31 |
| 170 | SRR17234890 | 65.59 | 99.351 | 1188620 | 4465117 | 40 |
| 171 | SRR17234903 | 65.6 | 99.36 | 4420657 | 4420657 | 24 |
| 172 | SRR17231610_subreads | 65.63 | 99.368 | 4441154 | 4441154 | 46 |
| 173 | SRR9176614 | 65.6 | 99.382 | 4454921 | 4454921 | 53 |
| 174 | SRR26139251 | 65.6 | 99.395 | 4014319 | 4429202 | 19 |
| 175 | ERR904400 | 65.57 | 99.417 | 609643 | 4399965 | 3 |
| 176 | SRR17234888 | 65.63 | 99.436 | 480956 | 4514930 | 54 |
| 177 | SRR9176753 | 65.6 | 99.44 | 4451688 | 4451688 | 50 |
| 178 | SRR17231462_subreads | 65.64 | 99.454 | 4445905 | 4445905 | 53 |
| 179 | SRR26282632 | 65.62 | 99.464 | 2979839 | 4449640 | 34 |
| 180 | ERR3427694 | 65.65 | 99.472 | 4462129 | 4462129 | 44 |
| 181 | SRR26282613 | 65.62 | 99.489 | 2670145 | 4437636 | 40 |
| 182 | SRR26282631 | 65.61 | 99.514 | 4110149 | 4451127 | 43 |
| 183 | SRR17231410 | 65.66 | 99.589 | 4456809 | 4456809 | 47 |
| 184 | SRR26282625 | 65.62 | 99.604 | 4441775 | 4441775 | 41 |
| 185 | SRR17234895 | 65.6 | 99.616 | 4438987 | 4438987 | 37 |
| 186 | SRR26139252 | 65.61 | 99.7 | 4421936 | 4421936 | 18 |
| 187 | ERR10561256 | 65.69 | 99.757 | 4430273 | 4430273 | 5 |
| 188 | ERR904411 | 65.61 | 99.77 | 899493 | 4423138 | 2 |
| 189 | ERR904403 | 65.61 | 99.882 | 1521848 | 4418431 | 3 |
| 190 | ERR904396 | 65.63 | 99.887 | 2623832 | 4428117 | 2 |
| 191 | ERR904402 | 65.62 | 99.9 | 2263394 | 4432286 | 3 |
| 192 | ERR904408 | 65.61 | 99.925 | 1982287 | 4422345 | 2 |
| 193 | SRR9626913_subreads | 65.62 | 99.969 | 4425622 | 4425622 | 6 |
| 194 | ERR904405 | 65.62 | 99.984 | 2032230 | 4431583 | 3 |
| 195 | ERR904404 | 65.62 | 99.986 | 4423470 | 4423470 | 2 |
